# Aerobic Training Associated with Arginine Supplementation Reduces Collagen-Induced Platelet Hyperaggregability in Rats under High Risk to Develop Metabolic Syndrome

**DOI:** 10.1155/2019/8919435

**Published:** 2019-01-06

**Authors:** Nadia A. V. Motta, Milla M. Fumian, Renata F. Medeiros, Gabriel F. Lima, Christianne B. V. Scaramello, Karen J. Oliveira, Antonio C. L. Nóbrega, Fernanda C. F. Brito

**Affiliations:** ^1^Laboratory of Experimental Pharmacology (LAFE), Department of Physiology and Pharmacology, Biomedical Institute, Fluminense Federal University (UFF), Room 204-A, 24420-210 Niterói, RJ, Brazil; ^2^Department of Physiology and Pharmacology, Fluminense Federal University (UFF), 24420-210 Niterói, Rio de Janeiro, Brazil

## Abstract

**Background:**

Increased platelet response is seen in individuals with metabolic syndrome. Previous reports have shown that arginine supplementation and aerobic exercise training enhance vascular nitric oxide (NO) activity and inhibit platelet hyperaggregability; however, the effects of their association remain unknown.

**Aim:**

To investigate whether arginine supplementation and aerobic exercise association may exert beneficial effects, reducing platelet hyperaggregability in rats under high risk to develop metabolic syndrome.

**Methods:**

Wistar rats were divided into two groups: control (C) and fructose (F – water with 10% of fructose). After two weeks, the F group was subdivided into four groups: F, the same as before; fructose + arginine (FA – 880 mg/kg/day of L-arginine by gavage); fructose + training (FT); and fructose + arginine + training (FTA). Treatment lasted for eight weeks.

**Results:**

The fructose administration was able to increase the collagen-induced platelet aggregation (27.4 ± 2.7%) when compared to the C group (8.0 ± 3.4%). Although the arginine supplementation (32.2 ± 6.3%) or aerobic training (23.8 ± 6.5%) did not promote any change in platelet collagen-induced hyperaggregability, the association of arginine supplementation and aerobic exercise promoted an inhibition of the platelet hyperaggregability induced by fructose administration (13.9 ± 4.4%) (*P* < 0.05). These effects were not observed when ADP was employed as an agonist. In addition, arginine supplementation associated with aerobic exercise promoted a decrease in interleukin-6 (IL-6) and interleukin-8 (IL-8) serum levels when compared to the fructose group, demonstrating an anti-inflammatory effect.

**Conclusions:**

Our data indicate an important role of arginine supplementation associated with aerobic exercise, reducing platelet hyperaggregability and inflammatory biomarker levels in rats under high risk to develop metabolic syndrome.

## 1. Introduction

Platelets are small, anucleated cell fragments that are activated in response to a wide variety of stimuli, triggering a complex series of intracellular pathways leading to a hemostatic thrombus formation at vascular injury sites. Reported abnormalities in platelet functions, such as platelet hyperactivity and hyperaggregability to several agonists, contribute to the pathogenesis and complications of thrombotic events associated with hypertension [1]. Metabolic syndrome (MS) represents a collection of cardiovascular risk factors: hypertension, hyperglycemia, dyslipidemia, and abdominal obesity. More than 312 million adults worldwide have been diagnosed with MS, and it is estimated that this number will rise to 1 billion by 2025 [2]. MS is linked to chronic inflammation. Normal physiological coagulation is altered when the inflammatory mediators modify the endothelium in the damaged area which becomes proinflammatory and prothrombotic [3]. Increased platelet response is seen in individuals with MS, supported by the main features of MS: insulin resistance, hyperglycemia, dyslipidemia, oxidative stress, products of adipose tissue (adipokines), and inflammation [4]. In hypercholesterolemic rabbits and humans, arginine administration enhances vascular nitric oxide activity and inhibits platelet reactivity [5, 6]. L-Arginine is also known to directly inhibit platelet reactivity, probably through its metabolism to nitric oxide (NO) by platelet-derived nitric oxide synthase (NOS) [6]. It is well known that an exercise conditioning program results in a downregulated hemostatic potential by reducing platelet activation, diminishing procoagulant platelet levels and increasing fibrinolysis activity, therefore contributing to a reduced risk of thrombotic events [7, 8]. High fructose consumption has been correlated to the onset of cardiometabolic alterations in world population. Chronic fructose ingestion has been used as a model of cardiovascular disease, characterized by increased serum insulin and triglyceride levels as well as vascular disorder development [9]. Some mechanisms associated with arginine supplementation and aerobic training effects on different cardiovascular disease models have been described, but their effects at platelet aggregation remain unclear. Thus, our hypothesis is that arginine supplementation and aerobic exercise may exert beneficial effects on MS development through reducing platelet hyperaggregability.

## 2. Material and Methods

### 2.1. Experimental Design

The handling and experimental protocols were approved by the Ethics Committee for Care and Use of Laboratory Animals at Fluminense Federal University (protocol 466/2013) and complied with the ethical guidelines of the Brazilian Society of Laboratory Animal Science. Before beginning the experimental protocol, all animals were submitted to an adaptation period for 4 weeks and then we started the protocol. Experiments were performed on 3-month-old male Wistar rats (*n* = 7/group; 329.9 ± 8.9 g). The experimental model has been previously described by our group [9]. Animals were maintained under controlled temperature conditions (24 ± 1°C) on a 12-hour light cycle (lights on at 7 a.m.).

**Figure 1:**
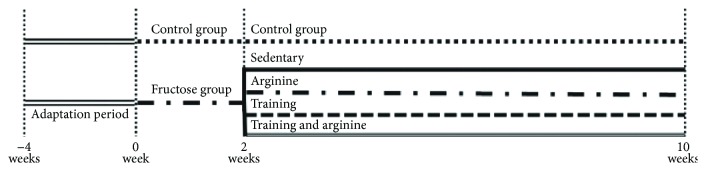
Experimental protocol.

Animals were randomly allocated, initially, into two groups: a control group (C) that received water and commercial chow for two weeks and a fructose group (F) that received an overload of 10% of D-fructose in drinking water and commercial chow for two weeks. After two weeks, seven animals from each group were euthanized, and the remaining animals from the control group (*n* = 7) were kept in the same previously described conditions for an additional eight weeks. Rats from the fructose group were subdivided into four further groups, and all of them continued receiving D-fructose in drinking water for an additional eight weeks. The fructose arginine (FA) group received 880 mg/kg/day of arginine by orogastric gavage. The fructose training (FT) group was submitted to aerobic exercise training, and the fructose training arginine (FTA) group was subjected to a combination of aerobic training with arginine supplementation (Figure 1).

### 2.2. Dietary Assessment

All experimental groups were maintained under the same commercial chow (Nuvilab Cr-1®, NuVital, Paraná, Brazil) *ad libitum*, according to Table 1. Caloric intake was assessed twice weekly, and it was calculated by considering that 1.0 g of chow corresponds to 3.36 kcal and 1.0 g of fructose corresponds to 4.0 kcal.

Fructose groups received 10% diluted D-fructose (Sigma-Aldrich, St. Louis, MO, USA) in water *ad libitum* [10]. The L-arginine-supplemented groups received 880 mg/kg/day (Sigma-Aldrich, St. Louis, MO, USA) for the last 8 weeks; this dose was calculated according to the formula for dose translation based on body surface area [11], considering 10 g for an adult person [12]. In this context, it is important to notice that this amino acid is naturally found in human nutrition in edible mushrooms [13] and in meals as beef, pork, and poultry [14].

### 2.3. Aerobic Exercise Protocol

Before beginning the experimental protocol, all animals were submitted to an adaptation period on a treadmill (Inbrasport®, Brasília, Brazil) for 4 weeks (5 minutes/day; 0.3 km/h–1.0 km/h, increased weekly). All animals underwent a maximal exercise test (MET) on a treadmill with an 11% inclination and an initial speed of 1.0 km/h, with an increment of velocity of 0.1 km/h every two minutes. The MET protocol was performed before the beginning of the experiment, two weeks thereafter, and at the end of the experiment to determine maximum running speed [9].

The groups assigned to aerobic training initiated a moderate-intensity exercise training regimen (50–75% maximal running speed), with a 0 to 7% inclination on a treadmill 4 days per week during the last 8 weeks of the experiment protocol [9]. All animals allocated to sedentary groups, in order to maintain their adaptation to the treadmill, were submitted to five minutes in the treadmill with low intensity (0.3 km/h), once a week.

At the end of the experimental period, all the animals were euthanized by cervical dislocation under anesthesia (thiopental sodium, 80 mg/kg) (Sigma-Aldrich, St. Louis, MO, USA). The blood samples were collected from each animal.

### 2.4. Biochemical Analysis

Serum lipid profile (levels of total cholesterol (TC), triglycerides (TG), low-density lipoprotein cholesterol (LDL), and high-density lipoprotein cholesterol (HDL)) was determined by using standard assay kits (Labtest, Minas Gerais, Brazil). The units were expressed in mg/dl.

### 2.5. Determination of Lipid Peroxidation in Serum Samples

The mean concentration of malondialdehyde (MDA), a measure of lipid peroxidation, was assayed in the form of thiobarbituric acid-reacting substances (TBARS) [15].

### 2.6. Platelet Aggregation Studies

The rat blood was removed by cardiac puncture and was collected into tubes containing a 3.8% trisodium citrate (9 : 1 *v*/*v*) solution. The rat platelet-rich plasma was prepared by centrifugation at 250 × *g* for 10 min at room temperature. The platelet-poor plasma was prepared by centrifugation of the pellet at 1500 × *g* for 10 min at room temperature.

Platelet aggregation was monitored by the turbidimetric method described by Born and Cross [16], using a platelet lumi-aggregometer (Model 560CA; Chrono-log Corporation, Havertown, PA, USA).

The platelet-rich plasma (400 *μ*l) was incubated at 37°C for 1 min with continuous stirring at 1200 rpm. The aggregation of the platelet-rich plasma was induced using platelet agonists as adenosine diphosphate (ADP) at (0.5 and 1 *μ*M) and collagen at (0.5 and 1 *μ*g/ml) (Sigma-Aldrich). Platelet aggregation was expressed as percentage of aggregation in response to ADP or collagen.

### 2.7. Interleukin-6 (IL-6) and Interleukin-8 (IL-8) Measurements

The serum levels of IL-6 and IL-8 were assessed using the commercially available Quantikine Rat IL-6 and IL-8 Immunoassay. The levels of IL-6 and IL-8 in the serum were assessed by measuring the absorbance at 450 nm using an ELISA reader (Tp Reader, Thermo Plate®) and extrapolating from a standard curve.

### 2.8. Statistical Analysis

The five experimental groups were compared using one-way ANOVA followed by a post hoc Bonferroni multiple-comparison test, when appropriate. All variables are expressed as means ± SEM. For all analyses, a value of *P* < 0.05 was considered to be statistically significant. All analyses were performed using the GraphPad Prism 5.0 software (GraphPad, San Diego, CA, USA).

## 3. Results

As described in Table 2, no significant difference in body weights of the five experimental groups was observed through the experimental period. Evaluating the serum levels of total cholesterol and LDL, we could not notice any statistical difference among the groups. However, an increase in serum HDL levels associated with the training (13.2 ± 1.1 × 21.9 ± 2.9 mg/dl) when compared to the Fructose group was observed, as well as a decrease in MDA concentrations in the serum of the training group (14.8 ± 1.5 × 9.6 ± 0.9 nmol/dl) (Table 2).

**Figure 2:**
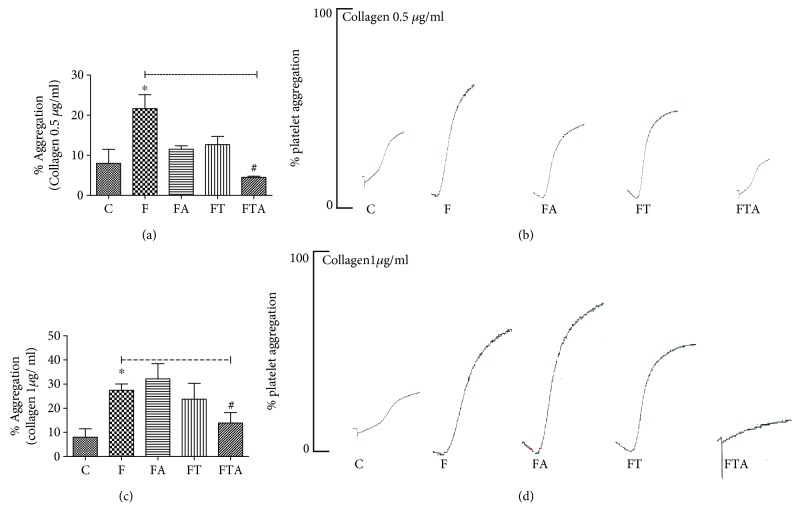
Platelet aggregation induced by collagen 0.5 *μ*g/ml (a, b) and collagen 1 *μ*g/ml (c, d) after 8 weeks of fructose overload. C group: control group; F group: fructose group; FA group: fructose + arginine group; FT group: fructose + training group; FTA group: fructose + training + arginine group. Data are presented as means ± SEM. Statistical analysis (one-way ANOVA and post hoc Bonferroni multiple-comparison test): ^∗^*P* < 0.05*v.* the C group and ^#^*P* < 0.05*v.* the F group.

Evaluating the fructose administration effects in collagen-induced platelet aggregation (0.5 *μ*g/ml), we could evidence an enhancement of platelet aggregation (27.4 ± 2.7%) when compared to the C group (8.0 ± 3.4%). Arginine supplementation (32.2 ± 6.3%) or aerobic training (23.8 ± 6.5%) was not able to promote any change at the platelet hyperaggregability. On the other hand, arginine supplementation associated with aerobic exercise promoted an inhibition in the platelet hyperaggregability induced by fructose administration (Figures 2(a) and 2(b)). Similar results were observed at 1 *μ*g/ml collagen concentration (Figures 2(c) and 2(d)).

**Figure 3:**
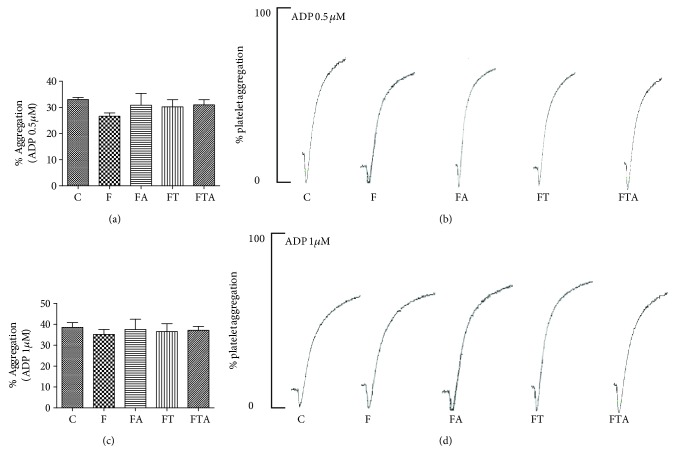
Platelet aggregation induced by ADP 0.5 *μ*M (a, b) and ADP 1 *μ*M (c, d) after 8 weeks of fructose overload. C group: control group; F group: fructose group; FA group: fructose + arginine group; FT group: fructose + training group; FTA group: fructose + training + arginine group. Data are presented as means ± SEM. Statistical analysis (one-way ANOVA and post hoc Bonferroni multiple comparison test): ^∗^*P* < 0.05*v.* the C group and ^#^*P* < 0.05*v.* the F group.

We have also evaluated the effects of fructose administration in platelet aggregation ADP-mediated (0.5 *μ*M and 1 *μ*M). When ADP was employed as an agonist, we could not notice any effect at the different employed conditions (Figure 3).

**Figure 4:**
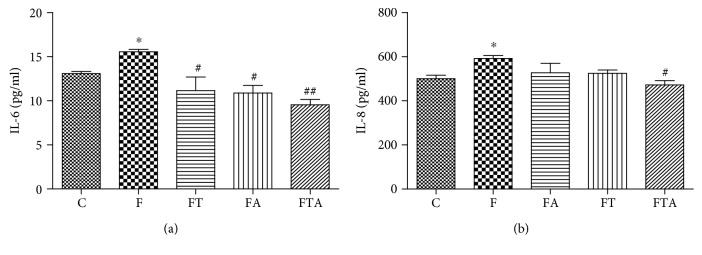
Effects of fructose intake, arginine supplementation, and aerobic exercise on IL-6, and IL-8 production in serum. C group: control group; F group: fructose group; FA group: fructose + arginine group; FT group: fructose + training group; FTA group: fructose + training + arginine group. Data are presented as means ± SEM. Statistical analysis (one-way ANOVA and post hoc Bonferroni multiple comparison test): ^∗^*P* < 0.05*v.* the C group ^#^*P* < 0.05 and ^##^*P* < 0.01*v.* the F group.

In our study, we have observed that fructose administration promoted a significant increase in IL-6 (15.87 ± 0.35 pg/dl) and IL-8 (592.40 ± 12.69 pg/dl) levels when compared to the control group (IL-6: 12.96 ± 1.60; IL-8: 500.20 ± 15.91 pg/dl). Concerning IL-6 serum levels, we have observed that arginine supplementation (10.89 ± 0.87 pg/dl) and aerobic exercise (IL-6: 11.16 ± 1.15 pg/dl) alone as well as associated (9.56 ± 0.61 pg/dl) reduced these interleukin levels. When we evaluated IL-8 levels, only arginine supplementation associated with aerobic exercise was able to reduce IL-8 levels (472.10 ± 19.41 pg/dl). There was no difference between arginine supplementation and aerobic exercise isolated when compared to the fructose group (*P* > 0.05, Figure 4).

## 4. Discussion

The present study was designed to investigate the antiplatelet effects associated with arginine supplementation, aerobic exercise, and these two concomitant interventions in rats at high risk of developing metabolic syndrome.

In our study, we have observed an increase in serum HDL levels and a decrease in MDA concentrations in the serum of the training group. These results corroborate the work of Farah et al. [17], which demonstrated that moderate aerobic exercise training prevented unfavorable changes in oxidative stress profile in rats submitted to a fructose overload and also the Ranjbar et al. [18] study, which described that L-arginine appears to have additive effects on cardiac function but has no effect on oxidative stress indices.

Evaluating the fructose administration effects at collagen-induced platelet aggregation, we could evidence an enhancement of platelet aggregation when compared to the C group. The arginine supplementation or aerobic training was not able to promote any change at the platelet hyperaggregability. On the other hand, arginine supplementation associated with aerobic exercise promoted an inhibition of the platelet hyperaggregability induced by fructose administration. We have also evaluated the effects of fructose administration at ADP-induced platelet aggregation. When ADP was employed as an agonist, we could not notice any effect at the different experimental groups.

Our data suggest that high fructose consumption results in an enhanced platelet response to collagen. This effect is reverted through the association of arginine supplementation with aerobic exercise. Interestingly, we could not observe this hyperaggregability effect when ADP is employed as an agonist, and the different treatments isolated were not able to have any antiplatelet effect. These results point out the antiplatelet effects when arginine supplementation is associated with aerobic exercise. Our research group [19] showed a vasodilator activity associated with an improvement of endothelial function and an increased NO bioavailability, observed in the group that received arginine supplementation, as well as in the aerobic exercise group treated with fructose. On the other hand, no additive effect in vascular relaxation in the interventions associated was observed [19].

Several studies have shown that IL-6 and IL-8 are important mediators of inflammation and contribute to the development of cardiovascular diseases. Independently of body mass index, sedentary lifestyle is a risk factor [20] and a strong predictor [21] for chronic disease and premature mortality. These proinflammatory cytokines exert a key player in the pathogenesis of cardiometabolic diseases [22] and thrombogenesis [23]. Additionally, activated platelets induce endothelial secretion of IL-8 in vitro. These data provide an important relationship between inflammation and cardiovascular events. In our experimental model, we observed that fructose intake was able to increase serum inflammatory mediators such as IL-6 and IL-8, corroborating to previous reports that demonstrated an increase in these cytokine levels in several pathological conditions [24–26]. In our experimental conditions, we observed that arginine supplementation, aerobic exercise, and the two associated interventions were able to reduce serum IL-6 levels. Concerning IL-8, only arginine supplementation associated with aerobic exercise reduced these proinflammatory marker levels, which was similar to our results observed in the platelet aggregation study. These observations are in line with previous work which reinforces that exercise plays an advantageous role in thrombogenesis by reducing inflammatory processes and potentiating fibrinolytic features [27], besides highlighting L-arginine's role in inflammatory cytokine inhibition through modulation of nuclear factor kappa B (NF-*к*B) [28].

Collagen is a component of the subendothelium which becomes exposed to flowing blood in the context of vascular injury. Collagen binds directly to two platelet surface receptors: integrin *α*_2_*β*_1_ and glycoprotein (GP) VI. Collagen also binds to the von Willebrand factor under shear, which binds to the GPIb-IX-V complex on platelets, resulting in vascular tethering and initiates signal transduction [29]. Collagen evokes a stable aggregation in rat platelets, and the main signaling pathways involved are ADP secretion and thromboxane A2 (TXA_2_) production [30]. Kobzar et al. [31] demonstrated that short-term exposure of platelets to monosaccharides as glucose, fructose, and galactose impairs inhibition of platelet aggregation by cyclooxygenase inhibitors like aspirin. These authors aimed to explain the reason why aspirin treatment is not effective in reducing cardiovascular events in diabetic patients. They suggested that lactic acid produced by anaerobic glycolysis in platelets might be a mediator of the effect of monosaccharides on aspirin inhibition in platelets, probably through an enhancement of arachidonic acid production.

There is evidence that spontaneous platelet aggregation was significantly higher in MS patients compared with healthy volunteers. The curves of the mean aggregate sizes and light transmission characteristics suggested that the rates of collagen-induced aggregation of isolated platelets in MS patients significantly exceeded the corresponding values in the group of healthy volunteers [32].

Patients with MS have high risk of microcirculation complications and microangiopathies. Inflammation influences coagulation by increasing the production of coagulation proteins, reducing the activity of the anticoagulant pathway and by preventing fibrinolysis. Together, these alterations could lead to the formation of pathological thrombi resulting in heart or brain infarcts. The presence of MS could affect the coagulation system in some way before atherosclerosis development [33].

ADP is considered a weak agonist which promotes platelet aggregation through activation of purinergic receptors [34]. Collagen represents a strong platelet agonist, activating different signal pathways. Our results demonstrated collagen-induced platelet hyperaggregability in rats under high risk of developing metabolic syndrome. These contribute to better understanding of the role of platelet alterations in MS development.

**Figure 5:**
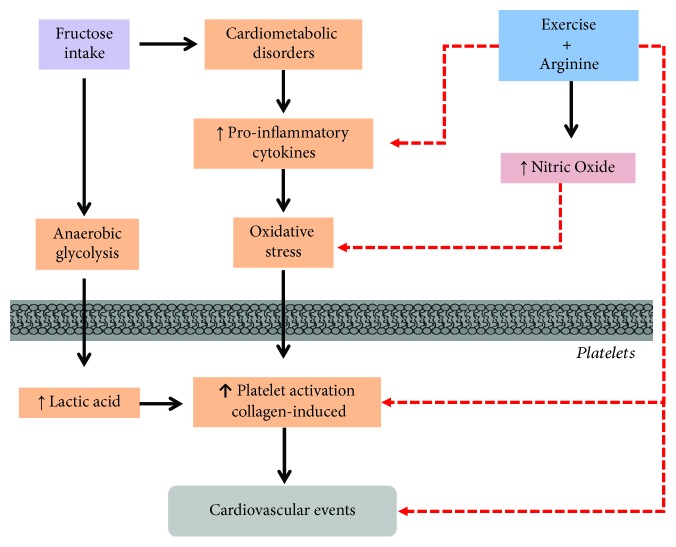
The hypothesis of inhibitory effect of exercise and arginine association in platelet activation of rats under high risk to develop metabolic syndrome. The high fructose intake triggers several cardiometabolic disorders, reflecting in an increase in lactic acid, proinflammatory cytokines, and oxidative stress. These events contribute to collagen-induced platelet activation. In addition, the increase in lactic acid produced by anaerobic glycolysis in platelets might be a mediator in platelet hyperaggregability. On the other hand, aerobic training associated with arginine supplementation decreases platelet hyperaggregability collagen–induced probably related to enhancement of NO production, inhibition of proinflammatory cytokines and oxidative stress, and finally inhibition of platelet aggregation.

Interestingly, the association of aerobic exercise and arginine supplementation abolished this effect, but none of the isolated conditions presented any antiplatelet effect. These data support the idea that aerobic training associated with arginine supplementation decreases collagen-induced platelet hyperaggregability in an experimental model, with a continued exposure to a causal factor of metabolic alterations, therefore preventing cardiovascular disease development. The antiplatelet effect observed is probably related to the association of an enhancement of NO production and a reduction in oxidative stress and inflammatory cytokines, resulting in a reduction in platelet TXA_2_ production and platelet activation and aggregation (Figure 5). Similar results were observed by our group in vascular beds [9, 19].

## 5. Conclusion

High fructose intake leads to cardiometabolic alterations that precede cardiovascular disease. High fructose administration enhanced platelet aggregation and arginine supplementation associated with aerobic exercise can reduce platelet hyperaggregability in rats under high risk to develop metabolic syndrome.

Since a large number of individuals that are affected by MS suffer cardiovascular events, finding new therapeutic targets by elucidating new factors that contribute to these incidents is crucial to the treatment and prevention of cardiovascular events.

## Figures and Tables

**Table 1:** Nutritional information of Nuvilab Cr-1 commercial chow.

Nutritional information	1 kg of chow
Calories	3.360 kcal
Carbohydrates	530.0 g
Proteins	220.0 g
Lipids	40.0 g

*Ingredients*	
Ground whole corn, soybean meal, wheat bran, calcium carbonate, dicalcium phosphate, sodium chloride, vitamins A, D3, E, K2, B1, B6, B12, niacin, calcium pantothenate, folic acid, biotin, chloride choline, iron sulfate, manganese monoxide, zinc oxide, calcium sulfate, sodium selenite, cobalt sulfate, lysine, methionine, and butylated hydroxytoluene.	

Nutritional information and ingredient composition were obtained from chow label.

**Table 2:** Evaluation of body weight, lipids, and MDA serum levels.

Parameters evaluated	Experimental groups				
	C	F	FA	FT	FTA
Initial body weight (g)	334.5 ± 3.2	334.3 ± 3.1	311.7 ± 15.1	332.9 ± 11.3	336,0 ± 11.7
Δ weight (g)	83.2 ± 3.5	85.1 ± 4.2	85.5 ± 3.7	86.3 ± 4.4	81.3 ± 3.1

Total cholesterol (mg/dl)	30.5 ± 5.7	51.0 ± 4.7	32.3 ± 6.6	57.2 ± 5.8	44.4 ± 14.6
LDL (mg/dl)	22.1 ± 4.0	26.2 ± 9.4	19.7 ± 3.5	28.4 ± 12.0	27.0 ± 9.8
HDL (mg/dl)	11.2 ± 0.5	13.2 ± 1.1	14.4 ± 1.7	21.9 ± 2.9^∗^^#^	17.8 ± 1.5
MDA (nmol/dl)	11.2 ± 0.7	14.8 ± 1.5	14.3 ± 1.1	9.6 ± 0.9^∗^^#^	17.3 ± 2.0

Data are presented as means ± SEM. Δ weight (g) = (final body weight − initial body weight). Statistical analysis (one-way ANOVA and post hoc Bonferroni multiple-comparison test): ^∗^*P* < 0.05*v.* C group; ^#^*P* < 0.05*v*. F group.

## Data Availability

The platelet aggregation data used to support the findings of this study are partially included within the article, but all the platelet aggregation registers and spectrophotometric registers used to support the findings of this study are available from the corresponding author upon request.
